# Programmed Cell Death-Ligand 1 (PD-L1) Immunohistochemical Expression in Equine Melanocytic Tumors

**DOI:** 10.3390/ani14010048

**Published:** 2023-12-22

**Authors:** José Pimenta, Justina Prada, Isabel Pires, Mário Cotovio

**Affiliations:** 1 CECAV—Veterinary and Animal Research Center, University of Trás-os-Montes e Alto Douro, 5000-801 Vila Real, Portugal; jprada@utad.pt (J.P.); ipires@utad.pt (I.P.); mcotovio@utad.pt (M.C.); 2 Associate Laboratory for Animal and Veterinary Sciences (AL4AnimalS), 5000-801 Vila Real, Portugal; 3 CIVG—Vasco da Gama Research Center, EUVG—Vasco da Gama University School, 3020-210 Coimbra, Portugal; 4 Veterinary Sciences Department, University of Trás-os-Montes e Alto Douro, 5000-801 Vila Real, Portugal; 5 Faculty of Veterinary Medicine, Lusófona University, Campo Grande 376, 1749-024 Lisbon, Portugal

**Keywords:** equine, melanocytic tumors, PD-L1, immunotherapy

## Abstract

**Simple Summary:**

Programmed cell death-ligand 1 (PD-L1) is expressed by several tumors, promoting tumoral immunosuppression by binding to programmed cell death protein (PD-1). PD-1/PD-L1 blockade in human melanoma has been shown to result in tumor regression and prolonged tumor-free survival. Since there are only a few available treatments for equine melanoma, the search for new therapies is important. This work intended to study the immunolabeling of PD-L1 in equine melanocytic tumors. A total of 77 melanocytic tumors were classified as benign or malignant and evaluated by extension of labeling. A total of 59.7% of the tumors showed >50% of immunolabeled cells. Regarding malignant tumors (*n* = 38), 24 tumors presented >50% of labeled cells, 13 tumors presented between 25–50% and one tumor presented <10%. Regarding benign tumors (*n* = 39), 22 tumors presented >50% of labeled cells, nine tumors presented 25–50%, three tumors presented 10–25%, two tumors presented <10% and three tumors did not present expression. The results of this study suggest that PD-L1 may have therapeutic potential for equine melanomas.

**Abstract:**

Currently available treatments for equine melanocytic tumors have limitations, mainly due to mass localization and dimension, or the presence of metastases. Therefore, a search for new therapies is necessary. Programmed cell death-ligand 1 (PD-L1) is expressed by several tumors, blocking T cell-mediated elimination of the tumor cells by binding to programmed cell death protein 1 (PD-1). A novel therapeutic approach using PD-1/PD-L1 blockade in human melanoma resulted in tumor regression and prolonged tumor-free survival. This study aimed to evaluate the immunohistochemical expression of PD-L1 in equine melanocytic tumors. A total of 77 melanocytic tumors were classified as benign or malignant and evaluated by extension of labeling. A total of 59.7% of the tumors showed >50% of immunolabeled cells. Regarding malignant tumors, 24/38 tumors presented >50% of labeled cells, 13 tumors presented between 25–50% and one tumor presented <10%. Regarding benign tumors, 22/39 tumors presented >50% of labeled cells, nine tumors presented 25–50%, three tumors presented 10–25%, two tumors presented <10% and three tumors did not present expression. Our results suggest that PD-L1 blockade may be a potential target for immunotherapy in equine melanocytic tumors and that future clinical research trials into the clinical efficacy of the anti-PD-L1 antibody are necessary.

## 1. Introduction

Melanocytic tumors are the third most common tumor type in horses, after sarcoid and squamous cell carcinoma [1,2]. While these tumors can occur in horses of all colors, melanocytic tumors have a genetic predisposition linked to the grey coat color mutation, leading grey horses to have a disease prevalence up to 80%, mainly in geriatric animals [3,4]. A breed predisposition is also reported, likely because some breeds present higher numbers of grey horses, such as Arabians, Lipizzaners, Andalusians and Lusitanos [5,6,7].

The most intriguing fact about equine melanoma is its prolonged benign behavior, characterized by slow growing, low invasiveness and low metastatic rates [1,7,8,9,10,11,12]. However, if left untreated, mass growth can have significant health consequences due to the large dimensions that these tumors can acquire and due to the typical locations where they usually appear. Lips, eyelids, perianal area, prepuce, tail and parotid region are some of the sensitive areas where these tumors typically emerge in horses [6,13]. Intense mass growth in these areas will cause compression and can compromise some important physiological functions, creating wellbeing and life-threatening consequences. Furthermore, with time, some of these tumors can acquire more aggressive behavior, presenting local invasion and widespread metastasis [8,14,15,16,17,18,19,20,21]. Besides the health consequences of this disease for the individual, it also has a major impact on the breeding and sale of affected and genetically predisposed horses, thus having important economic consequences.

Over the years, a broad spectrum of treatment modalities has been used to control this tumoral disease in horses. The therapeutic options available can be divided into those with local tumor effect and those intended to prevent and cure systemic oncologic disease [13,22].

Local therapies such as surgery, intratumoral chemotherapy and intratumoral immunotherapy, among others, are the most common practices currently reported to treat equine melanocytic tumors [22]. Surgery is the most advisable approach for early and small masses, reporting success rates of 90% on the excised tumors. However, it has some limitations in cases where the location of the tumor is difficult to access and when the volume of the tumor makes total excision impossible. In addition, the healing time is quite long, the disease progression and continuous growth of non-treated tumors remains equal and it does not prevent the emergence of new tumors [8,22,23,24,25,26,27]. Intratumoral chemotherapy has success rates around 81%; however, its effectiveness is reported to be inversely proportional to the size of the tumor, meaning that it does not work so well in advanced disease [8,28,29]. Intratumoral immunotherapy, although promising, still fails to demonstrate significant and reproducible results. Furthermore, some of the reported immunotherapies have never been commercially available for clinical use [22,30,31,32,33,34,35,36].

Contrasting with the wide range of local therapies accessible for horse melanoma, there are only a few reported systemic options, all focusing on immunotherapy. The DNA vaccine Oncept^®^ is the most studied and used systemic immunotherapy against equine melanoma, although there are others reported. Once again, although some studies have made progress on this topic, recent literature shows mixed results and fails to show reproducible and significant results [13,22,30,37,38,39]. As such, to date, equine clinical practitioners remain limited mostly to local therapies to control disease consequences. 

The immune system has an important role in cancer progression. Tumors have numerous mechanisms to suppress anti-tumor immune responses, allowing immune evasion and disease progression. One of these mechanisms is the up-regulation of coinhibitory receptors, also known as immune checkpoints [40,41,42].

Programmed cell death-1 (PD-1) is an immune checkpoint expressed on T-cells and which regulates their action. Its ligand, programmed cell death ligand-1 (PD-L1), has already been shown to be expressed by many tumor types [41,43]. PD-L1-positive tumor cells have the capability to inhibit the action of tumor-infiltrating lymphocytes that express PD-1, via PD-1/PD-L1 binding. In both human and canine melanocytic tumors, PD-L1 expression has already been reported [44,45,46]. Furthermore, PD-1/PD-L1 blockade is an established therapeutic approach for human melanoma, with some anti-PD-1 antibodies (pembrolizumab, nivolumab, cemiplimab) and anti-PD-L1 antibodies (atezolizumab, durvalumab, avelumab) approved by the FDA, which demonstrates their ability to induce remission of the disease [47]. Regarding canine melanoma, studies on this therapy are scarce; however, there is already some evidence that supports PD-1/PD-L1 blockade as a potential therapy for this type of tumor. No severe adverse effects were reported after treatment, and tumor mass regression and longer survival time were observed even in dogs which had pulmonary metastasis [40,48,49,50,51,52].

Considering the existent evidence regarding the positive clinical effects of PD-1/PD-L1 blockade in the treatment of melanoma, it is pertinent to assess whether equine melanomas are potential candidates for this therapy. Although some articles have been published, according to the authors’ knowledge, the literature regarding PD-L1 expression in equine tumors is scarce and the sample dimension in the published articles is quite small, compromising the interpretation of the results [53,54,55].

The aim of this work was to study PD-L1 immunoexpression in equine melanocytic tumors, to evaluate whether it could be a potential target for immunotherapy in these animals. We also evaluate possible associations between PD-L1 expression and clinicopathologic features.

## 2. Materials and Methods

### 2.1. Tissue Samples

This study included primary formalin fixed paraffin-embedded samples from horses with a previous clinical and histological diagnosis of melanocytic tumors. 

### 2.2. Clinical Information 

The following information was collected from clinical reports: gender, coat color, mass localization, age, breed. Mass localization was evaluated individually and also grouped into cutaneous and mucocutaneous localization, to see if there were differences in PD-L1 expression. Not all clinical reports contained complete clinical information. Age was grouped into 3 categories: young: ≤5 years old; adult: between 6 and 14 years; geriatric: ≥15 years.

### 2.3. Histopathological Evaluation

Hematoxylin and eosin (HE)-stained sections were re-examined by two independent pathologists (IP, JP). After this evaluation, a bleaching protocol was applied to allow proper visualization of some histological features. This protocol consisted of incubation of the slides in 0.25% potassium permanganate for 1 h, followed by incubation in 2% oxalic acid for a maximum of 10 min (depending on the amount of pigment). This processing was controlled individually on each slide.

A division of tumors into malignant and benign melanoma was performed using the following histological features of malignancy [56,57]: tumor vascular emboli (present, absent), nuclear grade (I—when nuclei had minimal variations in shape and size compared to normal nuclei; II—moderate alterations on nuclear shape; III—irregular and larger than normal nuclei) and mitotic count (mitosis per ten high power fields (HPF)). Melanomas were considered as malignant if they presented any of the following: (i) tumor vascular emboli; (ii) more than 10 mitoses in 10 HPF or (iii) fewer than 10 mitoses but a nuclear grade of II or III. 

Tumors were eliminated if a reliable classification was not achieved or if the tumor did not resist the bleaching process.

The histopathological features considered by [56,57] were evaluated: degree of pigmentation (absent, slight, medium, high, very high); cell shape (epithelioid, spindle, mixed); circumscription (absent, present) and presence of epidermal ulceration (absent, present). 

### 2.4. Immunohistochemistry

The immunohistochemical technique was performed using a commercial detection system (NovoLink Polymer Detection System; Novocastra, Leica Biosystems, Newcastle, UK), according to the manufacturer’s instructions. 3 µm tissue sections were dewaxed in xylene and hydrated using a series of alcohol solutions, ending with tap water. Citrate buffer solution (0.01 M pH 6.0 ± 2) was used for microwave antigen-retrieval (1 cycle of 5 min at 750 W). After antigen retrieval, bleaching was performed. Endogenous peroxidase was blocked using 3% hydrogen peroxide for 5 min and endogenous protein blocking was also performed for 5 min. Primary antibody Anti-PD-L1 Antibody (ab233482, Abcam, Amsterdam, Netherlands) was diluted 1:200 in phosphate buffered saline (PBS) and incubated at 4 °C overnight. After this, slides were incubated with a secondary antibody. Immunostaining was visualized via incubation with 3,3′-diaminobenzidine tetrahydrochloride (DAB) chromogen. Slides were counterstained with Gill’s hematoxylin. The polyclonal antibody used reacts with human and mouse PD-L1. To evaluate the cross-reactivity of the antibody with horse species, the methodology reported by Benvegnen et al., 2021 [53] was used, where BLAST (https://blast.ncbi.nlm.nih.gov, accessed on 1 March 2023) revealed an 84.76% homology between the antibody amino acid sequence and equine PD-L1 amino acid sequence, which is highly predictive of cross-reaction.

### 2.5. Immunohistochemical Evaluation

**Figure 1:**
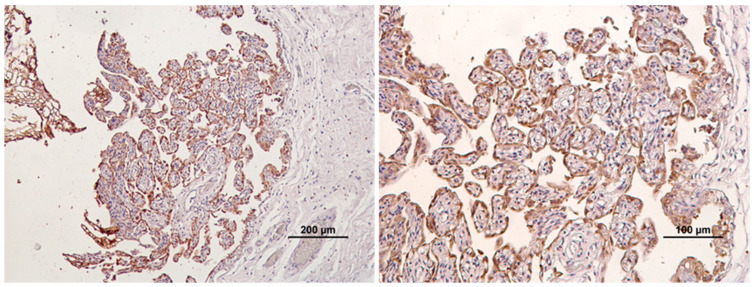
Positive control (equine placenta) with strong and diffuse PD-L1 immunolabeling.

A semiquantitative evaluation of PD-L1 staining was performed by two independent pathologists (IP, JP). Positivity was indicated by membranous or membranous and cytoplasmatic brown labeling. Cytoplasmatic labeling alone was considered nonspecific. Each staining run included a positive control (equine placenta) (Figure 1) and negative control (omission of primary antibody) [54].

PD-L1 staining was scored according to Benvegnen et al., 2021 as follows: 0—negative, (1) <10% labeled cells; (2) 10–25% labeled cells; (3) 25–50% labeled cells and (4) >50% labeled cells [53]. 

### 2.6. Statistical Analysis

To evaluate whether PD-L1 labeling was associated with histological classification (benign or malignant) or with histological/clinical features studied, the Chi-square (X^2^) test of independence and Fisher’s exact test were used. To compare medians of labeling extension between groups (benign and malignant or cutaneous and mucocutaneous localization), Mann–Whitney was used. Results were considered statistically significant when *p* < 0.05. Statistics were generated using Jamovi (version 2.3.2) statistical software.

## 3. Results

### 3.1. Clinical Information

The study sample included 57 horses, of which 27 were females and 28 were males. The average age was 14.3 ± 5.44 years (ranging from 2 years old to 26 years old). A total of 39 horses were geriatric (≥15 years old), 13 were adult (between 6 and 14 years old) and three horses were young (≤5 years old). The presented coat colors were grey (*n* = 46), cremello (*n* = 2), buckskin (*n* = 1) and brown (*n* = 1). Affected breeds were Pure-breed Lusitano (*n* = 27), Crossbreed (*n* = 19), Arabian (*n* = 3) and Warmblood (*n* = 2). The localizations of tumoral masses found were tail (*n* = 32), perianal region (*n* = 27), proximal limb (*n* = 2), lips (*n* = 2), vulva (*n* = 1), trunk (*n* = 1), parotid gland (*n* = 1) and neck (*n* = 1), which were grouped into cutaneous (*n* = 36) and mucocutaneous (*n* = 30). The tumor from the parotid gland was not considered to be cutaneous or mucocutaneous. 

### 3.2. Histopathologic Results

A total of 77 melanocytic tumors were included in our sample, which were divided into malignant (*n* = 38) and benign melanomas (*n* = 39).

**Figure 2:**
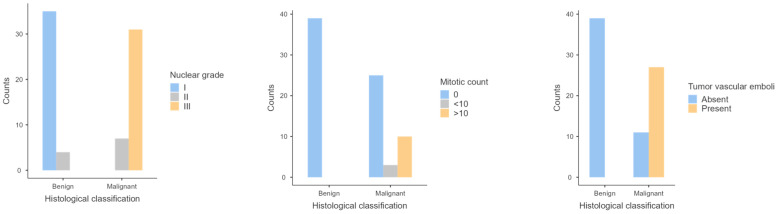
Graphical representation of the distribution of the histological features of malignancy (nuclear grade, mitotic count and tumor vascular emboli).

No association between histological classification and the histological features evaluated was reached, namely ulceration (*p* = 0.43), circumscription (*p* = 0.20), degree of pigmentation (*p* = 0.26) or cell shape (*p* = 0.18). However, the only amelanotic tumor of our sample presented all the histological features of malignancy evaluated, namely vascular emboli, >10 mitosis per 10 HPF, nuclear grade III and large nucleolar size. Table 1 contains the distribution of histological features of malignancy of the sample. This distribution is also represented in Figure 2.

Histological classification was not associated with clinical features either, namely coat color (*p* = 0.14), age (*p* = 0.184), breed (*p* = 0.83), gender (*p* = 0.23) and tumor localization (*p* = 0.35). Tumors belonging to non-grey horses presented all the histological features of malignancy. Most of the malignant tumors belonged to adult horses which were 10 or more years old (*n* = 15 tumors) and to geriatric horses (*n* = 20 tumors). Two of the horses included in the study presented more than one tumor mass (one horse had 13 benign melanomas and another horse had nine malignant melanomas). In each of these horses, the different tumoral masses were located in the same place, namely on the tail of the horse for benign melanomas and in the perianal region of the horse for malignant melanomas.

### 3.3. Immunohistochemical Results

**Figure 3:**
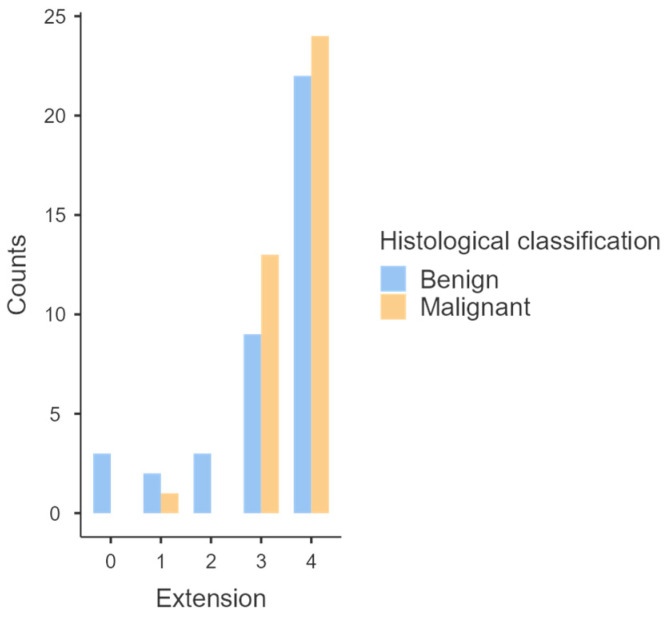
Graphical representation of the distribution of PD-L1 expression between benign and malignant melanomas.

**Figure 4:**
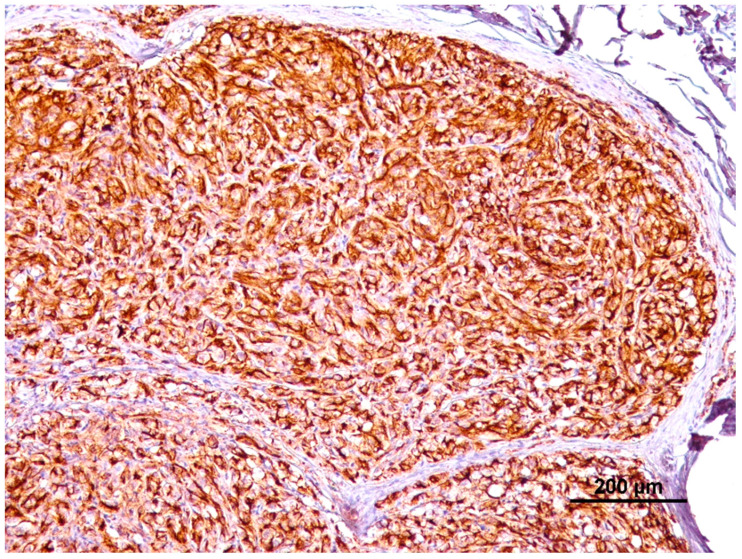
Diffuse PD-L1 extension of labeling (>50% of labeled cells) with membranous staining.

Results regarding extension of labeling are represented in Table 2 and Figure 3. Basically, 46 of 77 tumors (59.7%) presented more than 50% of labeled cells (Figure 4), which corresponds to 24/38 (63.2%%) malignant melanomas and 22/39 (56.4%) benign melanomas; 22/77 tumors (28.6%) presented between 25–50% of labeled cells, corresponding to 13/38 (34.2%) malignant melanomas and 9/39 (23.1%) benign melanomas; 3/77 tumors (3.9%) presented 10–25% of labeled cells, corresponding to 3/39 (7.7%) benign melanomas; 3/77 tumors (3.9%) presented <10% of labeled cells, corresponding to 1/38 (2.6%) malignant melanoma and 2/39 (5.1%) benign melanomas and only 3/77 (3.9%) tumors did not present any labeling, being all benign melanomas. PD-L1 expression did not differ between benign and malignant melanomas (*p* = 0.26). PD-L1 extension was not associated with any histological or clinical feature evaluated. In the case of horses that had multiple tumors, the individual tumors did not all present the same extension of labeling. 

**Figure 5:**
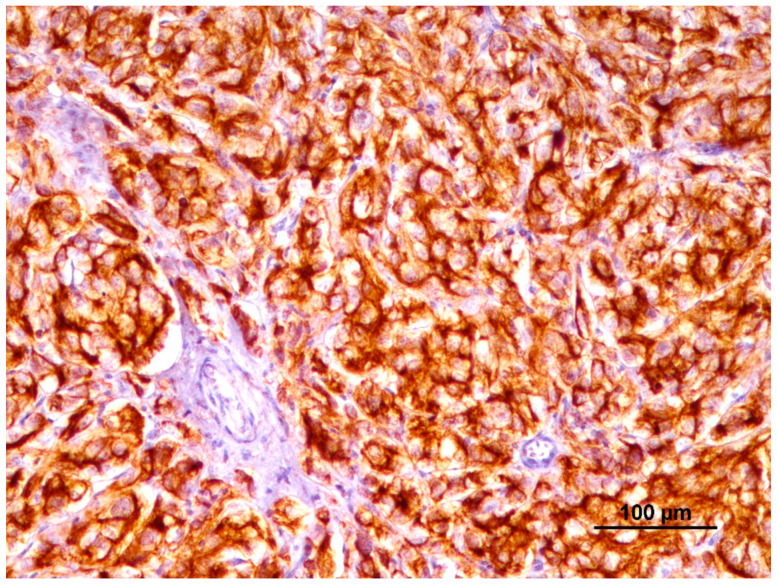
Diffuse membranous staining of tumor cells. Less frequent cytoplasmic staining can also be seen.

Regarding PD-L1 localization, all tumors presented tumor cells with membranous and cytoplasmatic labeling (Figure 5), being cytoplasmatic considered nonspecific. PD-L1 localization was not associated with any histological or clinical feature evaluated.

**Figure 6:**
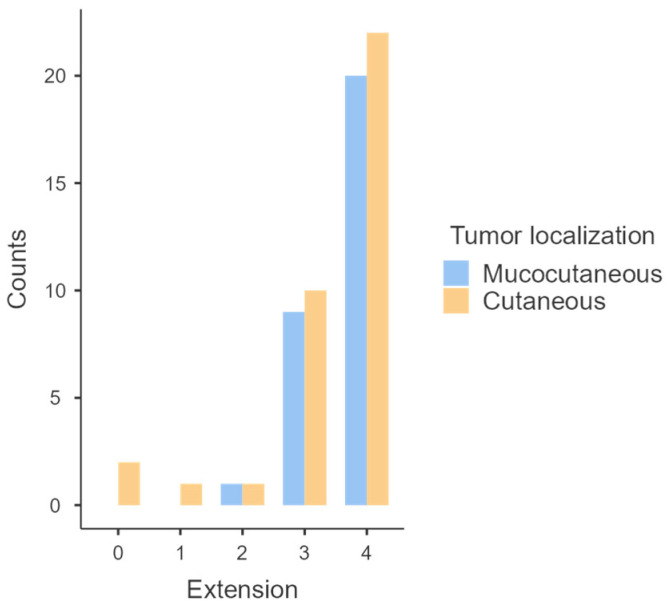
Graphical representation of the distribution of PD-L1 expression between cutaneous and mucocutaneous melanomas.

The results of PD-L1 expression in cutaneous and mucocutaneous tumors are presented in Table 3 and Figure 6. Basically, 22/36 (61.1%) cutaneous and 20/30 (66.7%) mucocutaneous tumors presented more than 50% of labeled cells; 10/36 (27.8%) cutaneous and 9/30 (30%) mucocutaneous tumors presented between 25–50% of labeled cells; 1/36 (2.8%) cutaneous and 1/30 (3.3%) mucocutaneous tumors presented 10–25% of labeled cells; 1/36 (2.8%) cutaneous tumor presented less than 10% of labeled cells and only 2/36 (5.6%) cutaneous tumors did not present any PD-L1 expression. PD-L1 expression did not differ between cutaneous and mucocutaneous localization (*p* = 0.68).

## 4. Discussion

Looking at therapeutic advances in other species could be a starting point for research into new solutions for equine melanocytic tumors. In this field, immunotherapy has been gaining ground, with immune checkpoint inhibitors being in the front line of cancer treatment. PD-1/PD-L1 blockade is one of the most recent immunotherapeutic strategies against human melanoma, presenting good response rates [41]. In veterinary medicine, great progress has been made in various canine tumor types, including melanoma [42,43]. However, the literature regarding these biomarkers in equine tumors is scarce and conclusions are difficult to draw due to the small samples used. According to the authors’ knowledge there is only one published article regarding PD-L1 in equine melanocytic tumors.

Ganbaatar et al., 2020 evaluated PD-L1 expression in four equine malignant melanomas, reporting that all samples presented immunolabeling [54]. Since few samples were included and no immunohistochemical score was provided, a proper comparison with our results is difficult to perform. Nevertheless, the high percentage (100%) of tumor immunolabeling was a promising preliminary result and correlates well with our work (96.1% of positive tumors). The same group also evaluated the in vitro effect of PD-1/PD-L1 blockade, reporting that cytokine production by peripheral blood mononuclear cells was enhanced, which could be a sign of immune cell activation. The results of PD-L1 evaluation are not so promising regarding equine sarcoids. Benvegnen et al., 2021 evaluated PD-L1 expression in nine equine sarcoids, reporting that 3/9 were negative, 4/9 had less than 10% of labeled cells and 2/9 had 25–50% of labeled cells [53]. Although the sample was also small, the results may indicate that the PD-1/PD-L1 pathway is unlikely to be a potential therapeutic target for these tumors. Similar results were obtained for equine squamous cell carcinomas by Porcellato et al., 2021, who studied PD-L1 expression in 17 tumors with only one (5.9%) presenting expression [55]. Once more, these results may also indicate that equine squamous cell carcinoma may not benefit from PD-1/PD-L1 blockade therapy. 

The advances in the study of canine melanocytic tumors are far superior, with several pre-clinical and clinical studies performed. Recent articles have showed that PD-1/PD-L1 is involved in canine melanocytic tumor development, with high PD-L1 expression being reported and the effect of PD-1/PD-L1 blockade already demonstrated in in vitro and in vivo studies [43,44,45,48,49,50,51,52,58,59]. Shosu et al., 2016 studied the immunohistochemical expression of PD-L1 in 15 malignant melanomas, reporting positivity in 73% (11/15). Maekawa et al., 2021 made the same evaluation in 20 oral melanomas, finding that 95% (19/20) were positive. Maekawa et al., 2016 also performed the evaluation of the same biomarker with 90% (36/40) positive oral melanomas. All these results are within the percentage we found in equine melanocytic tumors. Even in the face of some studies on human melanoma, our results seem promising, with Botti et al., 2017 showing that 61.8% (55/89) of melanomas presented PD-L1 expression [60]. 

In our study, no association was found between PD-L1 expression and tumor localization. This result is in accordance with some studies in dogs [43]; however, it differs from other studies where canine oral melanomas presented higher expression (100%) than cutaneous (33%) and ocular (0%) melanomas [52]. In humans, PD-L1 levels also seem to differ according to tumor localization, with cutaneous melanoma presenting the highest and the uveal melanoma the lowest expression [61]. These results may indicate that success rates of PD-1/PD-L1 inhibitors vary according to mass localization. Since the diversity of tumor localizations in our sample was not high, this may have influenced our results. However, in horses, some tumor localizations can limit the use of certain conventional therapies such as surgery. For example, melanomas in the parotid region are some of the most difficult to access surgically. In our study, we had one tumor sample in this location which had a high PD-L1 expression. In the future, this may possibly be an alternative therapy for these more complicated cases. 

PD-L1 expression was not equal across different tumors from the same horse. Such evidence should be taken into consideration since it could lead to different therapeutical efficacy between these tumors. The same fact is highlighted in human studies, where PD-L1 expression showed heterogeneity between tumors of the same individual [62]. 

Equine melanocytic tumors are considered more aggressive and tend to have worst prognosis in solid-color horses or in cases of amelanotic melanomas [6]. In our sample, we had four solid-color horses and all of them presented PD-L1 expression (two tumors with extension 4; one with extension 3 and one with extension 1). This fact highlights that, if it works, PD-1/PD-L1 blockade could be an alternative therapy for these horses in which conventional therapies have lower success rates. The same occurs for amelanotic tumors. Obviously, the limited number of cases only allows us to make assumptions and not draw concrete conclusions about this topic. 

The promising results of PD-L1 expression in equine melanocytic tumors should be evaluated considering the in vitro and in vivo PD-1/PD-L1 blockade studies performed in canine tumors. In vitro studies have already reported that anti-PD-1 and anti-PD-L1 antibodies can enhance cytokine production by tumor-infiltrating cells and the proliferation of peripheral blood mononuclear cells [48,50,52]. Pilot clinical studies have presented objective antitumor responses with a reduction of tumor burden and prolonged survival time and proved to be safe when tested against canine melanomas [48,50]. More recently, Pantelyushin et al., 2021 explored the in vitro potential of some FDA-approved human immune checkpoint inhibitors in dogs, and founded that Atezolizumab (an anti-PD-L1 antibody) was cross-reactive, blocked the PD-1/PD-L1 pathway and increased cytokine production. This is one more piece of evidence that supports the necessity of testing these drugs in veterinary oncology. All this evidence, combined with our results, underlines the rationale for further research on this immunotherapy in horses.

Regarding clinical information, most horses were geriatric (*n* = 39), which is in accordance with the existent literature, which states that equine melanocytic tumor development is an age-dependent event, presenting higher prevalence in older grey horses [1,3,7,63,64]. Most horses were grey (*n* = 46), which is in line with its etiology in the horse, related to a genetic trait that confers grey coat color [3,64]. Although the literature refers to a tendency for old grey horses to develop malignancy [21,64], in the present study no association between age and malignancy was found, which could be possibly explained by the absence of clinical information in some reports, which influenced the statistics. Debate still exists regarding the role of breed in this disease; however, some authors believe that breed is not a risk factor and that the emphasis given to some breeds is related to the higher prevalence of grey horses in those breeds [7,13]. Age, gender and mass localization were not associated with histological diagnosis. Although the first two are in accordance with the reality of canine melanomas, localization is an important feature to evaluate in dogs and can predict clinical behavior, with oral melanomas often being more aggressive [65]. 

Although no studies concerning this histological feature are available for horses, an association between pigmentation and histological classification would not be expected, since both benign and malignant melanomas in horses are characterized by being highly pigmented. As such, our result confirms our expectations. In dogs, pigmentation is not associated with malignancy either [65]. A common histologic feature of equine melanocytic tumors is the presence of various cell shapes in both benign and malignant tumors [20]. As such, we were not surprised by the absence of an association between histological classification and cellular shape. Superficial ulceration on equine melanomas is commonly found in tumors that present massive mass expansion, which occurs in both benign and malignant melanomas. As such, it was unlikely that we would find an association between histological classification and ulceration.

Independently of the enthusiastic results of PD-1/PD-L1 therapy, several authors have already reported tumoral resistances against this treatment. One of the mechanisms of resistance proposed by several articles is the up-regulation of cyclooxygenase-2 (COX-2) [60,66,67]. As such, due to the major role of COX-2 in the PD-1/PD-L1 pathway, future studies about COX-2 expression in equine melanomas would be interesting. 

Although discussion continues about whether PD-L1 tumor expression evaluation is a useful predictive biomarker in response to PD-1/PD-L1 inhibitors, several recent studies in humans and dogs have corroborated its use [46,68,69,70]. Tumors presenting PD-L1 expression have better responses than those without expression [43,52,68,69,71]. Combining this information with the high expression of PD-L1 found in equine melanomas further strengthens the possibility that equine melanomas could benefit from this therapy. Although three of the tumors from our sample did not present PD-L1 expression, the reasons for that remains unclear.

Besides membranous localization, PD-L1 staining can often result in cytoplasmatic and nuclear labeling, which are considered nonspecific and inadequate for predicting immunotherapy efficacy in human medicine [72]. Furthermore, it is stated that cytoplasmatic PD-L1 cannot be reversed with commonly used anti-PD-L1 antibodies [73]. A recently published article on canine melanoma also follows the same score methodology, where only membranous staining was considered positive [74]. However, other recent studies regarding PD-L1 in canine melanoma considered both localizations as positive staining and reported that cytoplasmatic labeling was the most common [45,48,51]. During clinical trials where the anti-PD-L1 effect was tested against canine melanomas, good clinical responses were obtained in cytoplasmatic stained tumors and no association was noted between staining pattern (membranous or cytoplasmatic) and clinical response [45,48,51]. Although only membranous staining was considered positive in our study, all the equine melanomas showed concomitant cytoplasmic staining. With these facts in mind, future studies will need to investigate the role of cytoplasmic PD-L1 in equine melanomas, and consider that, as in humans, its presence may not indicate that these tumors are good candidates for this immunotherapy.

The main limitations of this study are the lack of an in vitro evaluation of the efficacy of PD-L1 inhibition in equine melanomas and the absence of follow-up information that could help us understand how PD-L1 evolves over time and whether it would have prognostic value.

## 5. Conclusions

In the present study, we report high levels of PD-L1 expression in benign and malignant equine melanomas. These results point to the possibility of PD-1/PD-L1 blockade being a future treatment against these tumors and reinforce the importance of further in vitro and in vivo studies to assess the true efficacy of this therapy in horses.

## Figures and Tables

**Table 1:** Distribution of the histological features of malignancy.

Histological Classification	Nuclear Grade			Mitotic Count			Tumor Vascular Emboli	
	I	II	III	0	<10	>10	Absent	Present
**Benign**	35	4	0	39	0	0	39	0
**Malignant**	0	7	31	25	3	10	11	27

**Table 2:** Distribution of PD-L1 immunolabeling between benign and malignant melanomas.

Immunohistochemistry		Benign (*n* = 39)	Malignant (*n* = 38)	Total (*n* = 77)
**Extension**	0 (negative cells)	3	0	3
	1 (<10% cells)	2	1	3
	2 (10–25% cells)	3	0	3
	3 (25–50% cells)	9	13	22
	4 (>50% cells)	22	24	46

**Table 3:** Distribution of PD-L1 immunolabeling between cutaneous and mucocutaneous melanomas.

Immunohistochemistry		Cutaneous (*n* = 36)	Mucocutaneous (*n* = 30)
**Extension**	0 (negative cells)	2	0
	1 (<10% cells)	1	0
	2 (10–25% cells)	1	1
	3 (25–50% cells)	10	9
	4 (>50% cells)	22	20

## Data Availability

All new data (figures) are contained within this article.
